# Supplementary figures and images for: Superoxide dismutases maintain niche homeostasis in stem cell populations
Source: eLife. 2026 Mar 23;13:RP96446. doi: 10.7554/eLife.96446 (PMC13008353; doi:10.7554/eLife.96446)

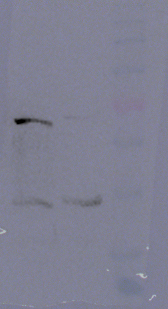

Supplement: Figure 2—figure supplement 1—source data 1. [file elife-96446-fig2-figsupp1-data1.zip › beta actin.Tif]

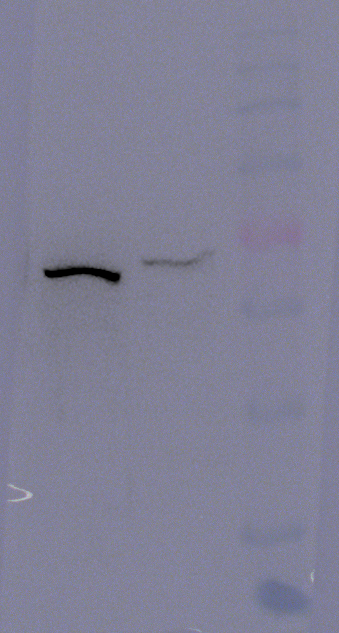

Supplement: Figure 2—figure supplement 1—source data 1. [file elife-96446-fig2-figsupp1-data1.zip › vasa.Tif]
