# Supplementary material for: Superoxide dismutases maintain niche homeostasis in stem cell populations
Source: eLife. 2026 Mar 23;13:RP96446. doi: 10.7554/eLife.96446 (PMC13008353; doi:10.7554/eLife.96446)
Supplement: Figure 2—figure supplement 1—source data 2. [file elife-96446-fig2-figsupp1-data2.zip › Western.pdf]

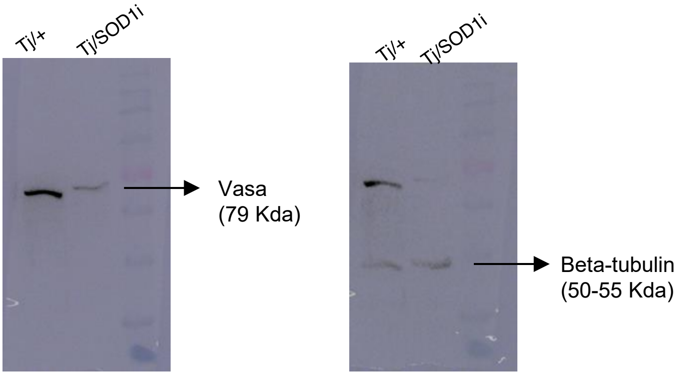

Figure 2, figure supplement 1N, source data1: Original blots corresponding to Figure supplement 1N. Beta tubulin is our internal control.
